# *CrI*_3_-*WTe*_2_: A Novel Two-Dimensional Heterostructure as Multisensor for *BrF*_3_ and *COCL*_2_ Toxic Gases

**DOI:** 10.1038/s41598-019-47685-5

**Published:** 2019-08-01

**Authors:** Amreen Bano, Jyoti Krishna, Tulika Maitra, N. K. Gaur

**Affiliations:** 1 Department of Physics, Barkatullah University, Bhopal, 462026 India; 2 Department of Physics, Indian Institute of Technology Roorkee, Roorkee, Uttarakhand 247667 India

**Keywords:** Two-dimensional materials, Electronic structure

## Abstract

A new multisensor (i.e. resistive and magnetic) CrI_3_-WTe_2_ heterostructure (HS) to detect the toxic gases BrF_3_ and COCl_2_ (Phosgene) has been theoretically studied in our present investigation. The HS has demonstrated sensitivity towards both the gases by varying its electronic and magnetic properties when gas molecule interacts with the HS. Fast recovery time (<0.14 *fs*) under UV radiation has been observed. We have considered two configurations of BrF_3_ adsorbed HS; (1) when F ion interacts with HS (C1) and (2) when Br ion interacts with HS (C2). In C1 case the adsorption energy *E*_*ad*_ is observed to be −0.66 eV while in C2 it is −0.95 eV. On the other hand in case of COCl_2_ *E*_*ad*_ is found to be −0.42 eV. Magnetic moments of atoms are also found to vary upon gas adsorption indicates the suitability of the HS as a magnetic gas sensor. Our observations suggest the suitability of CrI_3_-WTe_2_ HS to respond detection of the toxic gases like BrF_3_ and COCl_2_.

## Introduction

Gas detection for environmental monitoring has innumerable applications in the field such as industries and agriculture, medical diagnosis, military, etc.^1,2^ that utilizes the adsorption of gas molecules over materials. For the great technological perspective, it necessitates the material to have superior physical and chemical stability as well as the accessibility for chip-scale miniaturization of sensing elements for the low cost. Owing to the advent of 2D materials and increasing mass-market applications, the research in the gas sensors field have elevated rapidly due to a continuing need for the highly sensitive, selective and faster response and recovery dynamics towards gas adsorption. The first 2D atomic system ‘graphene’ has been enticed for a long time due to its extraordinary mechanical and electronic properties. The desired requirement of high surface area, carrier mobility, chemical and thermal stability with low electronic temperature noise, power consumption and higher response time promises graphene to be used in the next generation devices employed in gas sensing and bio-sensing^3–13^. Since each atom in graphene is a surface atom, it results in the ultrasensitive sensor response. It has been seen that the epitaxially grown graphene based sensors are ultrasensitive towards *NO*_2_ gas^14^. However, pristine graphene limits its potential upon physical adsorption of common gas molecules^15–18^ because of no dangling bonds. Thus for the chemisorptive enhancements, the surface is functionalized through polymers or metallic coating^19,20^. Other forms of the graphene like graphene oxide (GO) or reduced GO do serve as a dynamic material for high performance molecular sensors^21^. Inspired by the performance of the first 2D material, the gas sensing communities captured several hundreds of different 2D materials including elemental allotropes such as silicene, germanene borophene, etc., and compound like transition metal dichalcogenides (TMDs)^22^. These have been tremendously successful in detecting even the traces of gas molecules like *NO*_2_, *SO*_2_, *NH*_3_ etc.^23–26^. The forte of these materials is their ability to engineer artificial heterostructures (HS). Because of the van der Waals interactions between the HS, the lattice mismatching is not there that ultimately minimize the interfacial damages and chemical modification^27^.

Recently the integration between the magnetic layer and semiconductors initiate a new generation of advanced functional materials. These atomically thin magnetic materials are sensitive towards slightest of the perturbation (for instance by increasing the number of layers or changing the stacking order of the layers, by applying small magnetic field or by integrating with semiconductor) which could then influence the exchange coupling between neighboring spins. Thus, by manipulating exchange interactions in the magnetic materials, the electronic structure in 2D materials can be altered^28–30^. Integrating such thin magnetic materials with the non-magnetic semiconductor actually induces a two-sided effect: (1) magnetic material affects the magnetic property of the magnetic-semiconductor interface that ultimately modifies the electronic structure of semiconductor and, (2) the semiconductor affects the magnetism of the magnetic material. Such a combination of materials produces highly sensitive heterostructure that can alter its magnetic or electronic properties upon adsorption with foreign molecules. In this paper, we have investigated one such atomically thin magnetic material CrI_3_ monolayer integrated over WTe_2_ monolayer to form a CrI_3_-WTe_2_ heterostructure (HS) material.

The BrF_3_ is a hazardous gas used mainly in the processing of nuclear fuel. It is corrosive to metals and tissues and irritates the respiratory upon inhalation. It is a powerful oxidizer and highly reactive and a corrosive gas which can cause severe damage to the body like its contact can severely burn skin and eyes. On the other hand, phosgene is highly toxic gas used in industries for the production of pesticides and its immediate reaction starts even below 2–3 ppm. There are extensive reports on literature for COCl_2_ gas sensing and have shown better performance^31,32^. But, so far no investigation has been done on BrF_3_ gas adsorption on sensor layer. Thereby, it requires proper detection and thus necessitates a sensor that could do so.

Generally, the gas sensing mechanism is based on the principle of change in electronic properties with gas adsorption. Variation in magnetic properties upon gas adsorption has never been realized. Here we studied the gas sensing ability of a CrI_3_-WTe_2_ HS upon interaction with noxious gases BrF_3_ and phosgene (COCl_2_). Thus this paper focuses on the study of how the gas molecules (BrF_3_ and COCl_2_) interfere with the electrical and magnetic properties upon interaction. We have also investigated the nature of adsorption and selectivity towards each gaseous molecules. Practically, a sensor’s recovery time (*R*_*T*_) is crucial for technological applications, thus *R*_*T*_ for the highly selective gas molecule is calculated for this system.

## Computational Details

**Figure 1:**
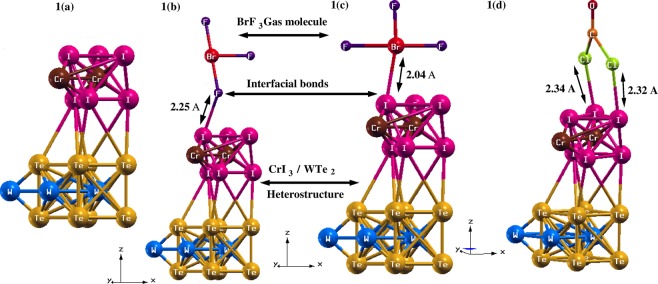
Crystal structures of (**a)** Pristine CrI_3_-WTe_2_ HS showing interfacial bonds among the parent compounds i.e. CrI_3_ and WTe_2_. These bonds result in net compression of the optimized HS. (**b)** BrF_3_ adsorbed CrI_3_-WTe_2_ HS with C1 configuration when F ion is directly interacting with the surface of the HS with a separation of 2.25 Å. (**c)** BrF_3_ adsorbed CrI_3_-WTe_2_ HS with C2 configuration when Br ion is directly interacting with the HS. The value of *d*_*eq*_ in this case has been observed to be 2.04 Å. (**d)** COCl_2_ adsorbed CrI_3_-WTe_2_ HS with *d*_*eq*_ in range 2.32 Å to 2.34 Å. Only one configuration is considered for COCl_2_ gas molecule due to the larger reduced mass of Cl as compared to O ion.

At ambient temperature and pressure conditions, the crystal structure of CrI_3_-WTe_2_ HS is shown in Fig. 1a. The results presented here are obtained using the first-principles approach which based on density functional theory^33^ as implemented in Quantum Espresso package^34^. Ideally, CrI_3_ exists in two crystal structures: (1) AlCl_3_ type monoclinic array and (2) BiI_3_ type rhombohedral order^35^. Here we report our findings for monoclinic assembly of CrI_3_ deposited over hexagonal structure of WTe_2_ ^27^. In order to explore the electronic structure of pure and BrF_3_/phosgene gas adsorbed CrI_3_-WTe_2_ HS, we have employed plane-wave ultrasoft pseudopotential method to trace the valance electron interactions. To serve the exchange-correlation potential, generalized gradient approximation (GGA) of Perdew-Burke-Erzernhof (PBE)^36^ has been implemented. A supercell of 2 × 2 × 1 has been used to construct CrI_3_-WTe_2_ HS. The cut-off kinetic energy of 760 eV has been applied with 7 × 7 × 1 K-mesh for Brillouin zones sampling. We have used these values after complete optimization process. To avoid any interaction among atomic orbitals we have provided a large vacuum of 17 Å along z-direction. The CrI_3_-WTe_2_ HS has been allowed to fully relax under the convergence of total energy and total forces which are found to be better than 1.0 meV. For gas sensing calculations, we have kept the structural geometry of CrI_3_-WTe_2_ HS fixed and periodically moved the gas molecules BrF_3_ and Phosgene COCl_2_ (one at a time) along z-direction in order to acquire the equilibrium distance *d*_*eq*_ between the HS and gas molecule. In case of BrF_3_ gas molecule, we have studied its interaction with the HS along two different orientations (1) F atom is interacting with HS surface and (2) Br atom is interacting with HS surface. The value of *d*_*eq*_ obtained in BrF_3_ in case 1 is ~2.25 Å whereas in case 2 it is observed to be ~2.04 Å. Moreover in case of Phosgene gas molecule *d*_*eq*_ is found within the range of 2.32 Å to ~2.34 Å. The adsorption energy of gas molecules BrF_3_ (in both cases) and Phosgene adsorbed over CrI_3_-WTe_2_ HS was defined as:

1 $${E}_{ad}={E}_{molecule/Cr{I}_{3}-WT{e}_{2}HS}-{E}_{Cr{I}_{3}-WT{e}_{2}HS}-{E}_{molecule}$$

where ${E}_{Cr{I}_{3}-WT{e}_{2}HS}$ and *E*_*molecule*_ indicates the total ground state energy of HS and gas molecule before adsorption take place respectively and ${E}_{molecule/Cr{I}_{3}-WT{e}_{2}HS}$ shows the total ground state energy of gas molecule adsorbed HS. It is worth to mention that the adsorption of gas molecules with the HS is attributed to surface adsorption hence the gas molecules will interact only with the CrI_3_ top surface layer.

## Results and Discussion

In the present investigation, we have studied the HS which is comprised of a monolayer of CrI_3_ deposited over the honeycomb WTe_2_ monolayer. Because of the presence of magnetic Cr^3+^ ion in CrI_3_ layer of HS, we have first carried out the calculation for two different magnetic configurations namely, ferromagnetic (FM) and antiferromagnetic (AFM) spin states at Cr site. Since the AFM state gives higher energy as compared to FM one, thus FM configuration is the stable magnetic state which is in accordance with the previous reports^35^. Hence, further investigations have been done for FM configuration only.

### Structural analysis

The pristine HS showed in Fig. 1a is composed of WTe_2_ and CrI_3_ monolayers. From Fig. 1a we can see that in WTe_2_, the W ions form a zig-zag pattern along a-axis resulting in slightly distorted hexagonal symmetry. The Te ions constitute an octahedral environment accompanied by strong intra-layer covalent bonding w.r.t. W ions. Whereas, in the CrI_3_ layer of HS, Cr^3+^ ions form a honeycomb lattice. The I- ions create an edge sharing octahedrally coordinated network w.r.t. Cr^3+^ ions such that the three I- ions are coordinated at the top and bottom layer of Cr ions. The two parent compounds (WTe_2_ and CrI_3_ monolayer) are vertically stacked together along c-axis to form a CrI_3_-WTe_2_ HS with interfacial bonds linking I and Te ions. The 〈*Te*−*I*〉 average bond length at the interface is 2.61 Å. An overall compression along c-axis has also been observed among the parent compounds of the HS which may affect its electronic structure. Table 1. displays the comparison of experimental and calculated bond length in HS. From the results obtained in Table 1 we observed a net compression in the HS (i.e. <45%) except for 〈*W*−*W*〉 (~11%) due to its relatively heavy atomic mass which obstructs any significant variation in its bond length as compared to other atoms. Hence the compression is emerging due to the interfacial bonds formed among the parent compounds of the HS (i.e. WTe_2_ and CrI_3_). These bonds are occurring from the charge transfer from Te ions to I ions (*charges flows from low electronegativity (Te* = *2.1 Pauling scale) to high electronegativity (I* = *2.6 Pauling scale)*). This process of bond formation at the interface of HS, in turn, results in compression of bond length among the atoms upon optimization. The electronic properties of the HS may get influenced due to this compression which has been discussed in detail in the following section. The interaction of BrF_3_ on HS can occur through two possible orientations: by forming an interfacial bond between (1) F and HS as shown in Fig. 1b (C1 configuration), (2) Br and HS as shown in Fig. 1c (C2 configuration). The 〈*F*−*Br*−*F*〉 bond angle is 86° with the 〈*Br*−*F*〉 bond length along an axial and equatorial plane as 1.72 Å and 1.81 Å respectively. The equilibrium distance (*d*_*eq*_) in C1 and C2 case is 2.25 Å and 2.04 Å respectively. For the COCl_2_ gas, the bond angle and bond length is 124° 〈*Cl*−*C*−*O*〉 and 1.76 Å 〈*Cl*−*C*〉, 1.19 Å 〈*C*−*O*〉 respectively. Unlike BrF_3_, only single orientation of COCl_2_ has been considered (Cl linked with HS) as presented in Fig. 1d due to the larger reduced mass of Cl w.r.t. O (about ~10%) ions. The *d*_*eq*_ varies from 2.32–2.34 Å for this case.

**Table 1:** Comparison of experimental and theoretically calculated bond length (Å) of parent compounds WTe_2_ and CrI_3_ in HS.

	Exp (Å)	Calculations (Å)
W-W^45^	3.6	3.2
W-Te^45^	2.769	1.448
Cr-Cr^35^	3.96	2.16
I-I^35^ (axis)	3.86	2.04
Cr-I^35^	2.72	1.43

The bond lengths from theoretical structure is lesser as compared to experiments which shows an overall compression in the HS. The net compression is due to the formation of interfacial bonds among the Te and I ions upon optimization.

### Electronic structure

**Figure 2:**
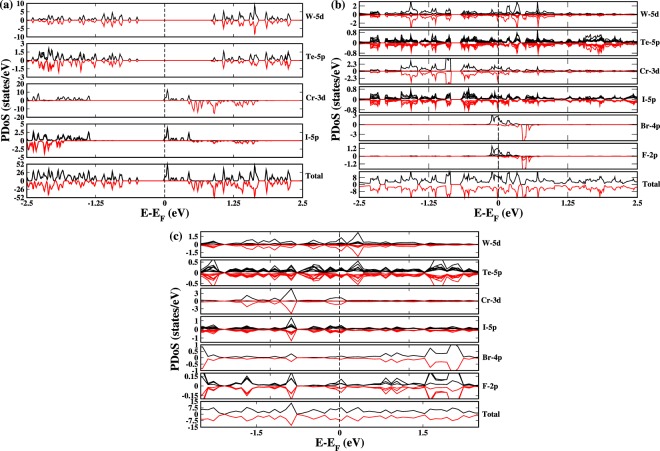
Results of Partial density of states (PDoS) of (**a**) Pristine CrI_3_-WTe_2_ HS. The total DoS of HS showing half-metallic nature which is coming from the Cr^3+^ ions having vacant 3d states. W-5d states and Te-5p states of WTe_2_ monolayer is showing an insulating character with a clear band gap of 1.31 eV. (**b)** C1 of BrF_3_ adsorbed HS. It is evident from the result that in C1 configuration the HS has become a metal, unlike pristine HS which is a half-metal. On the other hand, the spin-up states of the gas molecule BrF_3_ is actively contributing in the metallicity of the HS while spin-down states are present around 0.58 eV in the conduction band region. (**c)** C2 configuration of BrF_3_ adsorbed HS. It can be seen that when Br ion interacts with HS there is no spin splitting exists among both spin channels, unlike the C1 case.

In order to investigate the gas sensing effect of the HS, we have first studied the electronic density of states (DOS) prior to the gas adsorption. When no gas molecules were adsorbed, the total DOS of HS (Fig. 2a) shows a spectral weight of 11.83 states/eV at the Fermi level (FL). Small amount of metallicity is induced because of Cr-3d and I-5p orbitals of CrI_3_ layer of HS. This induced metallicity is emerging from the compressed bond length of the atoms upon optimization as discussed above. Whereas the WTe_2_ counterpart displays an insulating behavior with a gap of 1.31 eV between majority and minority spin channels. Experimentally, CrI_3_ layer is insulating in nature^35^ but in CrI_3_-WTe_2_ HS, half-metallicity is observed. This might be due to the electron doping of CrI_3_ layer induced by WTe_2_ as reported previously^37^. Near FL (E = −0.48 eV) only contributions from W-5d orbital and Te-5p orbital dominates whereas Cr-3d and I-5p orbital state lies 1.32 eV below FL. For pristine HS the total bandwidth for metallic state is observed to be 0.02 eV (Fig. 2a) with the majority spin carriers separated from minority spin carriers by 0.88 eV.

#### BrF_3_ adsorbed HS

**Figure 3:**
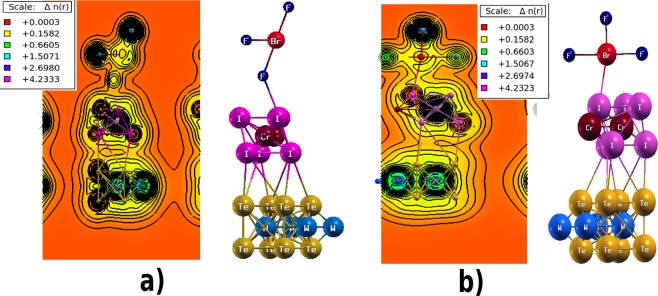
The charge density results of (**a**) C1 configuration of BrF_3_ adsorbed HS showing the charge transfer network among the HS and gas molecule. This also confirms the presence of chemisorptive nature of bond which has influenced the electronic structure of pristine HS. (**b)** C2 configuration of BrF_3_ adsorbed HS. The charges are appeared to flow from I ions to the gas molecule. Among all the atomic species present in the HS and the gas molecule, maximum electronegativity is possessed by F ions hence charges will get accumulate at F ions site.

When BrF_3_ gas molecule is adsorbed on the HS the metallicity is enhanced in both the orientations which can be seen from Fig. 2b,c. In C1 configuration (Fig. 2b), when F directly forms an interfacial bonding with HS, the bandwidth increases to 0.67 eV. At FL, the dominant contribution is coming from Cr-3d states with the spectral weight for up and down spin density being 0.85 states/eV and 1.56 states/eV respectively. Feeble participation of W-5d (up 0.33 states/eV, down 0.77 states/eV) and Te-5p (up 0.14 states/eV, down 0.21 states/eV) and I-5p states(up 0.23 states/eV, down 0.37 states/eV) are also observed at FL. The adsorbed BrF_3_ gas molecule in C1 have enhanced spin up DOS at FL. In principle, the charges should flow from Br (low electronegativity) to F (high electronegativity) ions but due to the large 〈*Br*−*F*〉 bond length (<1.7 Å) the charge hopping takes place at slower rate resulting in higher spectral weight of Br (2.08 states/eV) ion as compared to F (0.97 states/eV) ions at FL. On the other side for C2 configuration (Fig. 2c), when Br interacts directly with HS, the bandwidth further intensifies to 0.76 eV at FL. Likewise in C1, here also the Cr-3d states are pronounced at FL with spectral weight of 1.25 states/eV for all spin channels. Relatively weak involvement of W-5d (0.95 states/eV), Te-5p (0.2 states/eV) and I-5p (0.42 states/eV) states are present at FL. Now since the electronegativity of I, Br and F are 2.66, 2.96 and 3.98 Pauling scale respectively, therefore a continuous flow of the charge will take place from I to F via Br ion. Hence, net charge density at Br site decreases as compared to the previous case. In C2, the net charge transport takes place through I-Br-F chain which facilitates the smooth transfer without any accumulation while the charges stocked at Br site due to uneven path (I-F-Br) w.r.t. electronegativity in C1. To investigate the nature of bonding between BrF_3_ and HS, we have studied the charge density for both the orientations (Fig. 3a,b). For this purpose, the calculations were performed in (1 1 0) plane. As discussed earlier that the charge transport channel is decided by the electronegativity difference. In C1 (Fig. 3a), electronegativity of WTe_2_, CrI_3_ and BrF_3_ is 1.5071, 2.6980 and 4.2333 respectively where the maximum charge is accumulated near F (4.2333) ion of BrF_3_. Hence, a net flow of charge will take place from WTe_2_ to BrF_3_ layer via CrI_3_. Similar charge flow network is followed in C2 as shown in Fig. 3b where electronegativity of WTe_2_, CrI_3_ and BrF_3_ is observed to be 1.5067, 2.6974 and 4.2323.

#### Phosgene (COCl_2_) adsorbed HS

**Figure 4:**
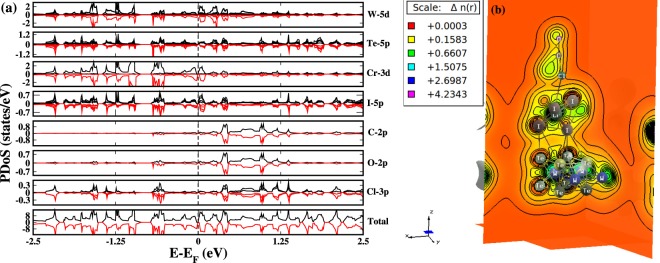
(**a)** PDoS of COCl_2_ adsorbed HS showing a ‘gapless’ type semiconducting behavior. The term gapless is justified by the fact that the electronic states of HS species are not overlapped at the Fermi level. The energy states of gas molecule COCl_2_ shows no significant contribution near the Fermi level. (**b)** The charge density result of Phosgene gas molecule adsorbed over HS indicates a unidirectional charge flow from HS to the gas molecule which causes the charge accumulation at COCl_2_. O ion is most electronegative in this case hence charges will be gathered at O ion site.

Another poisonous gas COCl_2_, when adsorbed on the HS surface, exhibits a gapless type semiconducting behavior which can be seen in Fig. 4a showing the PDOS of the above stated system. With adsorption, the states are present near the FL but do not cross for W and Te ions. A similar situation is observed for Cr-3d and I-5d states. This is in contrast with BrF_3_ adsorption where the metallicity was induced at each HS layer. Within −0.26 eV–0.31 eV energy window, the HS is occupied by the states of both spin channels near FL whereas, negligible contributions are coming from the gaseous states in that energy range. In the COCl_2_ gas molecule, the electronegativity of C, Cl and O are 2.55, 3.16 and 3.44 Pauling scale respectively. Thus, the charge flow from C will take place to O and Cl ions resulting in the slight occupation of states below FL in O and comparatively higher states at Cl site. The DOS of Cl ions (Fig. 4a) has the 3p states peaked within 0.5 eV–0.7 eV below FL due to higher charge density coming from C and HS layers. From the charge density calculations we observed a charge flow direction from HS to the gas molecule as given in Fig. 4b. The electronegativity of WTe_2_, CrI_3_ and COCl_2_ is 1.5075, 2.6987, 4.2343 respectively. It results in a unidirectional charge flow from bottom of HS to the gas molecule which causes the accumulation of carriers at COCl_2_. The above observations suggest that CrI_3_-WTe_2_ HS serve as a potential gas sensor for BrF_3_ and COCl_2_ gas molecule.

### Magnetic properties

In HS the magnetic contribution is coming due CrI_3_ layer which has FM ordering with the total spin magnetic moment of 5.35 *μ*_*B*_. And the magnetic moment per Cr and I ions are 2.86 *μ*_*B*_ and 0.041 *μ*_*B*_ respectively. This is in good agreement with the saturation moment(3.1 *μ*_*B*_/Cr) measured experimentally^35^. The low magnetic moment of I ions is due to the transfer of unpaired 4 s electron from Cr to I- resulting in stable 5p states. This delocalization of charges causes a reduced moment at I- site. On the other hand, the *WTe*_2_ layer in HS remains non-magnetic. With the exposure of *BrF*_3_ gas molecule in C1 configuration, the delocalization effects dominate in Cr ions resulting in decreased magnetic moment. The Br and F ions, on the other hand, acquire charges from HS have higher magnetic moment (0.088 *μ*_*B*_ for Br and 0.005 *μ*_*B*_, 0.144 *μ*_*B*_, 0.0268 *μ*_*B*_ per F ion) as compared to Cr^3+^ (0.009 *μ*_*B*_). As discussed above that due to the difference in 〈*Br*−*F*〉 bond lengths uneven charge flow takes places to form dissimilar magnetic moment per F ion. In C2 configuration when Br directly bonded with HS layer total magnetic moment is almost negligible. This is in accordance with the DOS of C2 (Fig. 2c) where the net reduction in spectral weight was observed. Due to the continuous charge transfer path (I-Br-F) the delocalization of electrons causes the moments to drop. The same scenario has been observed when Phosgene is exposed to HS. The overall reduction in the magnetic moment is observed here as well. We have tabulated (in Table 2) the % change in density of states of HS at Fermi level (Δ*ρ*) and magnetic moments (Δ*m*) with respect to no adsorption of gas for all the cases. The variation in magnetic moments of the HS upon the interaction of gas molecules suggests that CrI_3_-WTe_2_ HS can be used as a magnetic gas sensor as well as a resistive gas sensor. There are many studies over the latter type but only a few experimental studies are performed on the former type of gas sensor which detects the perturbation in the magnetic properties when gas molecules interact with the sensor material^38^. A few magnetic gas sensors so far studied are nanoparticles of *CuFe*_2_*O*_4_ which was used for the detection of volatile organic compounds (VOCs)^39^, Co/ZnO nanorods to detect *H*_2_ and CO molecules^40^, Co/ZnO hybrid nanostructures for the detection of *C*_3_*H*_6_*O*, CO and *H*_2_ target gases^41^ etc. Some of the experiments are mainly devoted to hybrid ferromagnetic/semiconducting materials such as Co/ZnO instead of common dilute magnetic semiconductors as the later requires high magnetic field for their application. In references (Ponzoni *et al*.^40^ and Ciprian *et al*.^41^) it has been found that in hybrid Co/ZnO system, the change in magnetization is linearly dependent on the concentration of gas. Thus, the higher the concentration of the gases, the higher would be the magnetization change. In our case, the system taken is also a hybrid one. In this we took a single gas molecule for the study, hence small changes in the magnetization have been observed.

**Table 2:** The table represents the % change in density of states (DOS) near Fermi level (Δ*ρ*) and % change in the magnetic moment (Δm) with respect to no adsorption of gas in the heterostructure (HS).

Gas adsorbed	Δ*ρ* (states/eV)	Δm (Bohr mag/cell)
**(a) BrF**_**3**_		
(i) Br interacts with HS	5.13	5.34
(ii) F interacts with HS	4.5 (spin up) and 2.97 (spin down)	0.64
(b) COCl_2_	gapless semiconductor	5.21

### Adsorption and recovery time

The adsorption energy describes the nature of stability among adsorbent (HS) and adsorbate (gas molecule). The process can take place in two modes (1) physisorption: which involves weak van der Waals forces between two reacting species. The electronic properties of adsorbent are barely perturbed during this mechanism; (2) chemisorption: here actual involvement of chemical bonds between species exists. This also requires minimum activation energy to initiate the process. In C1 with BrF_3_ adsorption on HS, the adsorption energy is −0.66 eV while in C2 it increases to −0.95 eV. The increasing adsorption rate by 30% suggests the comparatively strong chemisorptive nature in C2. The existence of strong covalent bonding between HS and *BrF*_3_ (C1 and C2) have been observed from charge density results shown in Fig. 3a,b. Similar studies for COCl_2_ adsorption shows the chemisorptive character but the *E*_*ad*_ (−0.42 eV) is relatively smaller than that from BrF_3_ interaction. It is evident that as *d*_*eq*_ increases, *E*_*ad*_ energy decreases. Thus, the higher *d*_*eq*_ for COCl_2_ case is marked by a decrease in *E*_*ad*_. Though the relative stability in COCl_2_ case is lesser than *BrF*_3_ but from previous literature COCl_2_ adsorption on BN nano tube (BNNT), BN nano rod (BNNR) and borophene reported to have *E*_*ad*_ −0.18 eV, −1.058 eV and −0.306 eV respectively^42,43^. Hence, COCl_2_ adsorption on *CrI*_3_-*WTe*_2_ HS has shown better performance as compared to previous reports with an exception of BNNR.

The recovery time *R*_*T*_ of a sensor is based on how fast the sensor retrieves its initial state. Based on the Arrhenius theory the sensor recovery time^44^ is related by:

2 $${R}_{T}={\nu }^{-1}{e}^{-{E}_{ad}/KT}$$

where *ν* is the operational frequency, *E*_*ad*_ is adsorbate energy, K is Boltzmann constant and T is the sensor’s operational temperature. For different attempt frequencies, the sensor’s recovery rate is affected as tabulated in Table 3. We have used one frequency each lying on IR, visible and UV range to calculate the corresponding R_*T*_ in each case. We believe that by changing frequency inside a specified range (ie., IR, visible and UV), the order of R_*T*_ will remain the same. The calculations are done for room temperature (300 K). Under UV illumination the HS is showing faster *R*_*T*_ for all the cases. The recovery rate depends on the nature of adsorption. With the relatively weak chemisorptive effect of *COCl*_2_ gas on HS, fastest recovery time (R_*T*_) is achieved.

**Table 3:** Adsorption energy (*E*_*ad*_(*eV*)) and recovery time *R*_*T*_ (fs) for BrF_3_ and COCl_2_ gas adsorption over HS layer.

	E _*ad*_ (*eV*)	*R*_*T*_ (IR) (fs)	*R*_*T*_ (Visible) (fs)	*R*_*T*_ (UV) (fs)
BrF_3_ (C1)	−0.66	1302	13.02	0.13
BrF_3_ (C2)	−0.95	1460	14.6	0.14
COCl_2_	−0.42	1180	11.8	0.12

In the I column, the *E*_*ad*_ of BrF_3_ and COCl_2_ gases showing chemisorptive nature. The highest *E*_*ad*_ in BrF_3_ represents the strongest covalent bonding between the gas molecule and HS. From the II, III and IV columns, the recovery is fastest when the sensor is illuminated with UV radiation.

## Conclusions

We have theoretically investigated a new 2-dimensional CrI_3_-WTe_2_ HS in the present work in order to explore the possibility as a multisensor (i.e. resistive and magnetic). Our results show that upon interaction with the gas molecules BrF_3_ and COCl_2_ with HS the electronic as well as magnetic properties of pristine HS get altered. We have also determined that how swiftly the HS can get recover after detaching the gaseous species from it by means of recovery time. We found that under UV illumination ultrafast recovery time is presented by the HS i.e. <0.14 *fs*. Hence we conclude that CrI_3_-WTe_2_ HS offers itself as a multisensor for the detection of highly toxic gases like BrF_3_ and COCl_2_.
